# Supplementary material for: hgtseq: A Standard Pipeline to Study Horizontal Gene Transfer
Source: Int J Mol Sci. 2022 Nov 22;23(23):14512. doi: 10.3390/ijms232314512 (PMC9738810; doi:10.3390/ijms232314512)

## Bos taurus

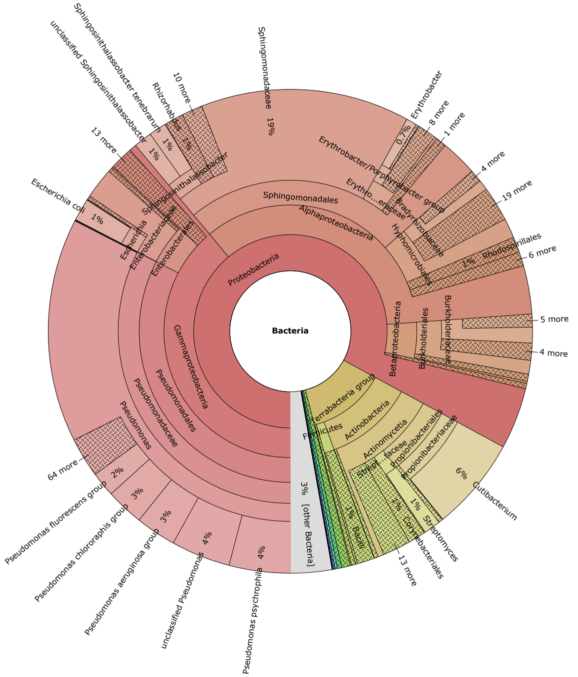


## Canis lupus familiaris

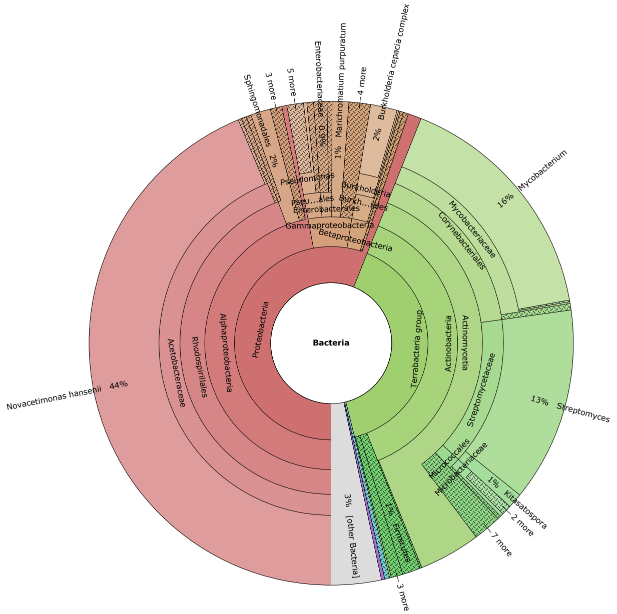


## Human CHIKV cohort

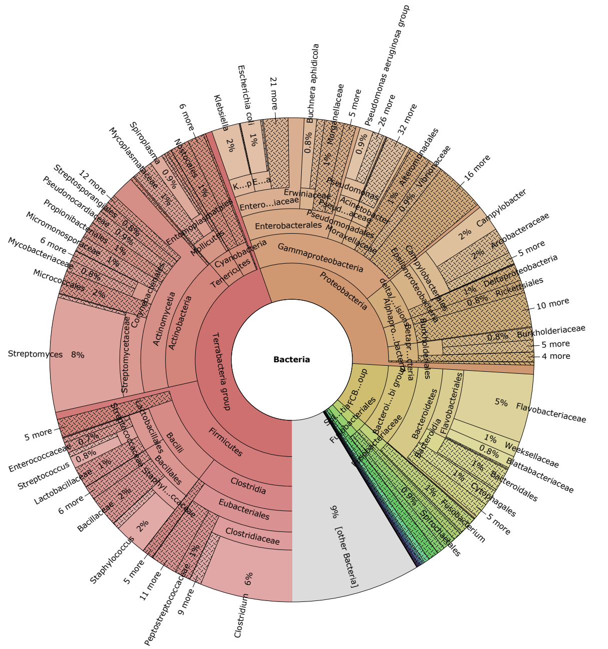


## Human CHIKV cohort

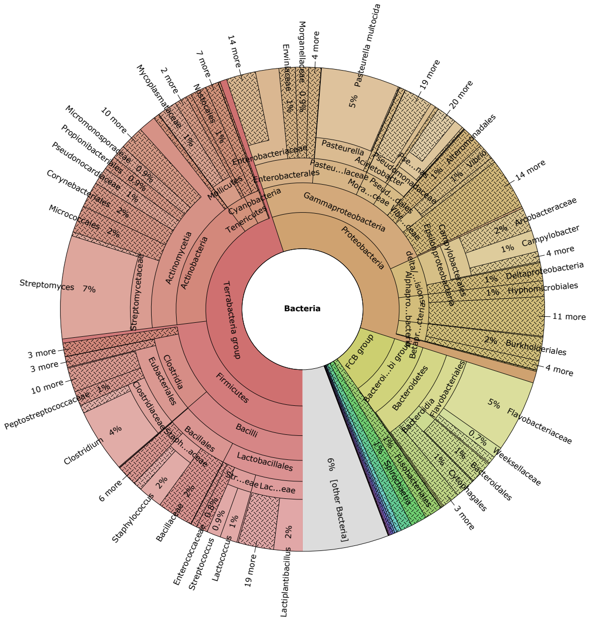


## Macaca fascicularis

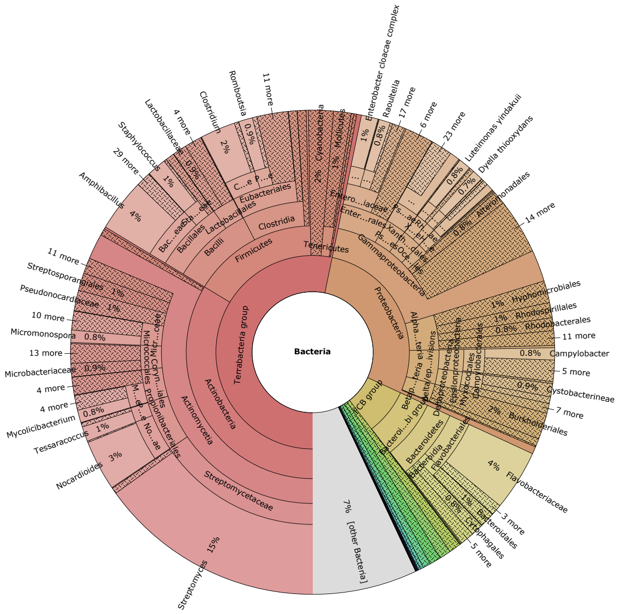


## Mus musculus

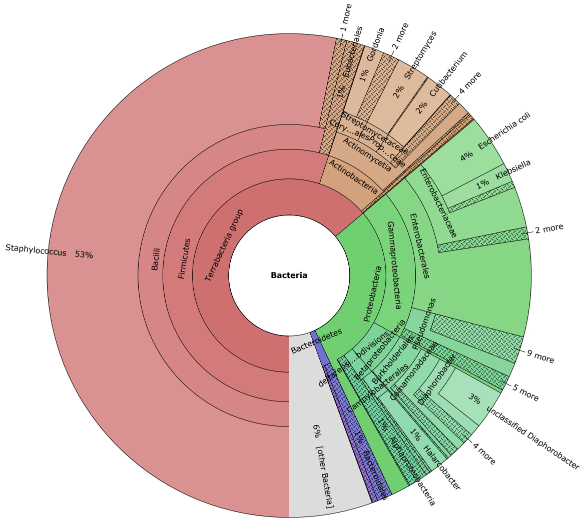

Supplement: Supplementary file 1 [file ijms-23-14512-s001.zip › Figure_S2.pdf]
