# Supplementary figures and images for: hgtseq: A Standard Pipeline to Study Horizontal Gene Transfer
Source: Int J Mol Sci. 2022 Nov 22;23(23):14512. doi: 10.3390/ijms232314512 (PMC9738810; doi:10.3390/ijms232314512)

**A**

## Single-unmapped reads

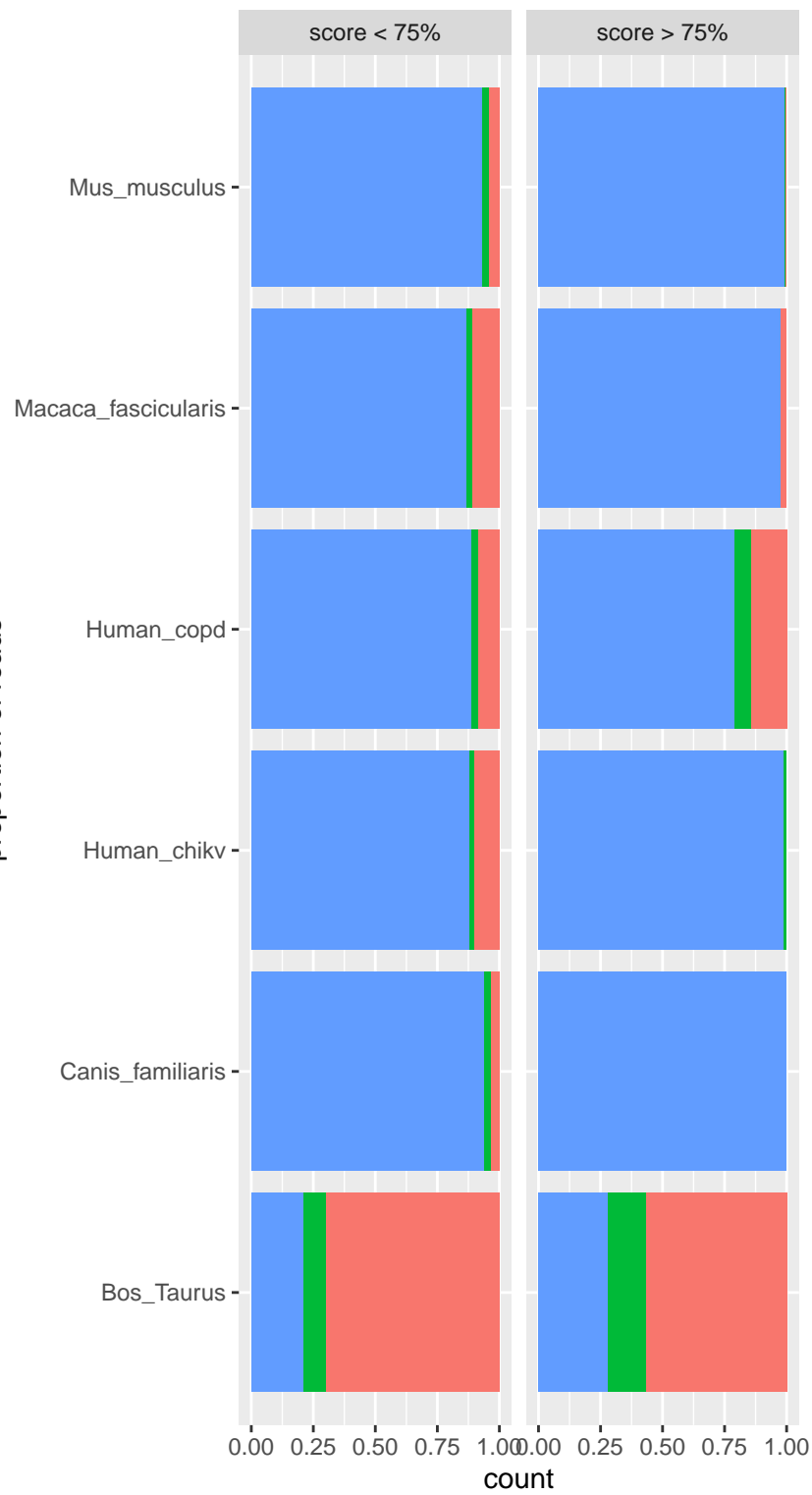**B**

## Both-unmapped

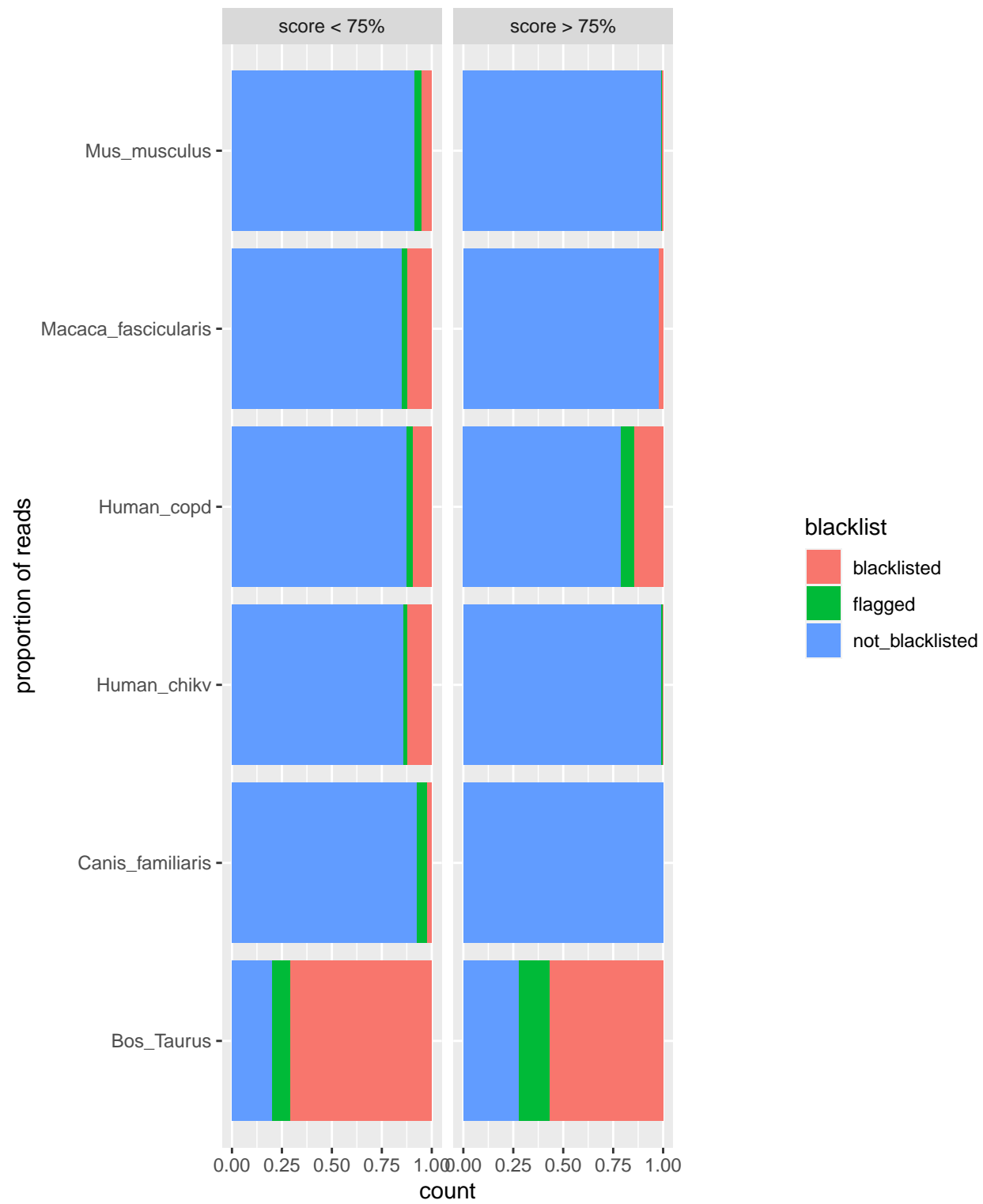

Supplement: Supplementary file 1 [file ijms-23-14512-s001.zip › Figure_S1.pdf]
